# Supplementary material for: Exploring Virulence Characteristics of Clinical Escherichia coli Isolates from Greece
Source: Microorganisms. 2025 Jun 26;13(7):1488. doi: 10.3390/microorganisms13071488 (PMC12300496; doi:10.3390/microorganisms13071488)
Supplement: Supplementary file 1 [file microorganisms-13-01488-s001.zip › microorganisms-3702111-supplementary.pdf]

Table S1. Characteristics of 100 *E. coli* isolates included in this study.

Isolate	Source	Department	ST	String formation	Hemolysin production	Biofilm production	Serum resistance	Resistance to antimicrobials	ESBL-encoding genes	CRISPR/Cas system
Eco-7827	Blood	Emergency medicine	10	No	Negative	Weak	Sensitive	None	None	-
Eco-7293	Urine	Emergency medicine	14	No	Negative	Weak	Sensitive	None	None	-
Eco-7702	Urine	Emergency medicine	58	No	Negative	Moderate	Sensitive	Amp, Amc, Sxt	None	Type I-E
Eco-3191	Urine	Neurology	69	No	Negative	High	Sensitive	Amp, Amc, Tzp, Fep, Caz, Mer, Amk, Gm, Cip, Sxt,	None	Type I-E
Eco-6371	Urine	Internal medicine	69	No	Negative	Moderate	Sensitive	Amp, Sxt	None	Type I-E
Eco-8539	Blood	Surgery	69	No	Negative	Moderate	Sensitive	Amp, Sxt	None	Type I-E
Eco-7686	Blood	Emergency medicine	95	No	Negative	Moderate	Sensitive	None	None	Type I-F
Eco-0648	Urine	Emergency medicine	104	No	Negative	Moderate	Sensitive	None	None	-
Eco-6119	Urine	Outpatient	104	No	Negative	Moderate	Sensitive	None	None	-
Eco-6257	Urine	Emergency medicine	104	No	Negative	Moderate	Sensitive	Amp, Amc	None	-
Eco-6338	Urine	Endocrinology	104	No	Negative	Moderate	Sensitive	None	None	-
Eco-7853	Urine	Emergency medicine	104	No	Negative	Moderate	Sensitive	Amp, Amc, Tzp	None	-

Eco-0352	Urine	Internal medicine	131	No	Negative	Moderate	Sensitive	Amp, Amc, Fep, Caz, Cip	*bla*CTX-M-1-like, *bla*CTX-M-9-like	-
Eco-2131	Blood	Internal medicine	131	No	Negative	Moderate	Sensitive	Amp, Amc, Fep, Caz, Cip, Sxt	*bla*CTX-M-1-like, *bla*CTX-M-9-like	-
Eco-2180	Blood	Emergency medicine	131	No	Negative	Moderate	Sensitive	Amp, Amc, Cip, Sxt	None	-
Eco-2201	Urine	Emergency medicine	131	No	Negative	Moderate	Sensitive	Amp, Amc, Tzp, Fep, Caz, Cip, Sxt	*bla*CTX-M-1-like, *bla*CTX-M-9-like	-
Eco-2254	Urine	Internal medicine	131	No	Negative	Moderate	Sensitive	Amp, Amc, Tzp, Fep, Caz, Cip, Sxt	*bla*CTX-M-1-like, *bla*CTX-M-9-like	-
Eco-2262	Urine	Urology	131	No	Negative	Moderate	Intermediate	Amp, Amc, Fep, Caz, Cip, Sxt	*bla*CTX-M-1-like, *bla*CTX-M-9-like	-
Eco-2264	Urine	Urology	131	No	Negative	Moderate	Sensitive	Amp, Cip, Sxt	*bla*CTX-M-9-like	-
Eco-2292	Urine	ICU	131	No	Positive	Moderate	Sensitive	Amp, Amc, Tzp, Fep, Caz, Amk, Gm, Cip, Sxt	*bla*CTX-M-1-like, *bla*CTX-M-9-like	-
Eco-2295	Unknown	Internal medicine	131	No	Negative	Moderate	Sensitive	Amp, Cip, Sxt	None	-
Eco-2309	Urine	Internal medicine	131	No	Negative	High	Sensitive	Amp, Amc, Fep, Caz, Gm, Cip	*bla*CTX-M-1-like	-
Eco-2423	Urine	Rheumatolo gy	131	No	Negative	Weak	Sensitive	Amp, Amc, Fep, Caz, Cip, Sxt	None	-
Eco-2424	Urine	Emergency medicine	131	No	Negative	Moderate	Sensitive	Amp, Amc, Fep, Caz, Cip	*bla*CTX-M-9-like	-
Eco-2425	Urine	Urology	131	No	Negative	Moderate	Sensitive	Amp, Amc, Tzp, Fep, Cip, Sxt	*bla*CTX-M-1-like	-
Eco-2434	Blood	Emergency medicine	131	No	Negative	Moderate	Sensitive	Amp, Fep, Caz, Gm, Cip, Sxt	*bla*CTX-M-1-like	-
Eco-2468	Blood	Surgery	131	No	Negative	Moderate	Sensitive	Amp, Amc, Tzp, Amk, Cip, Sxt	*bla*CTX-M-9-like	-

Eco-2470	Urine	Emergency medicine	131	No	Positive	Moderate	Sensitive	Amp, Amc, Fep, Caz, Gm, Cip, Sxt	*bla*$_{CTX-M-1}$-like, *bla*$_{CTX-M-9}$-like	-
Eco-2471	Urine	Emergency medicine	131	No	Positive	Weak	Sensitive	Amp, Amc, Tzp, Fep, Caz, Gm, Cip	*bla*$_{CTX-M-1}$-like, *bla*$_{CTX-M-9}$-like	-
Eco-2478	Urine	Emergency medicine	131	No	Negative	Moderate	Intermediate	Amp, Cip	*bla*$_{CTX-M-9}$-like	-
Eco-3087	Urine	Internal medicine	131	No	Negative	Moderate	Sensitive	Amp, Amc, Tzp, Fep, Caz, Amk, Cip	*bla*$_{CTX-M-1}$-like	-
Eco-3089	Urine	Urology	131	No	Negative	Moderate	Sensitive	Amp, Amc, Fep, Caz, Gm, Cip, Sxt	None	-
Eco-3091	Urine	Emergency medicine	131	No	Positive	Moderate	Sensitive	Amp, Amc, Fep, Caz, Gm, Cip	*bla*$_{CTX-M-1}$-like	-
Eco-3096	Blood	Internal medicine	131	No	Negative	Moderate	Sensitive	Amp, Cip, Sxt	*bla*$_{CTX-M-9}$-like	-
Eco-3106	Blood	Emergency medicine	131	No	Negative	Moderate	Sensitive	Amp, Amc, Tzp, Fep, Caz, Amk, Gm, Cip	*bla*$_{CTX-M-1}$-like	-
Eco-3110	Blood	Emergency medicine	131	No	Negative	Moderate	Sensitive	Amp, Amc, Tzp, Fep, Caz, Amk, Gm, Cip, Sxt	None	-
Eco-3174	Blood	Emergency medicine	131	No	Negative	High	Sensitive	Amp, Amc, Amk, Gm, Cip, Sxt	None	-
Eco-3190	Urine	Emergency medicine	131	No	Negative	Moderate	Sensitive	Amp, Amc, Tzp, Fep, Caz, Mer, Cip, Sxt	*bla*$_{CTX-M-9}$-like	-
Eco-3204	Blood	Emergency medicine	131	No	Negative	Moderate	Sensitive	Amp, Amc, Tzp, Fep, Caz, Mer, Amk, Cip	*bla*$_{CTX-M-1}$-like	-

Eco-6128	Sputum	Internal medicine	131	No	Negative	Moderate	Sensitive	Amp, Amc, Tzp, Cip	None	-
Eco-7274	Urine	Internal medicine	131	No	Negative	Weak	Sensitive	None	None	-
Eco-8282	Pus	ICU	131	No	Negative	Moderate	Sensitive	None	None	-
Eco-3090	Urine	Emergency medicine	140	No	Negative	Moderate	Intermediate	Amp, Amc	*bla*$_{CTX-M-9}$-like	-
Eco-2829	Blood	Internal medicine	156	No	Negative	Weak	Sensitive	Sxt	None	Type I-E
Eco-8593	Urine	Pediatrics	186	No	Negative	Moderate	Sensitive	Amp, Gm, Sxt	None	-
Eco-8623	Blood	Internal medicine	216	No	Negative	Weak	Sensitive	None	None	Type I-E
Eco-7461	Urine	Emergency medicine	390	No	Negative	Moderate	Intermediate	None	None	Type I-F
Eco-8617	Blood	Pediatrics	390	No	Negative	High	Sensitive	None	None	Type I-F
Eco-6289	Urine	Emergency medicine	410	No	Negative	Moderate	Sensitive	None	None	-
Eco-6502	Urine	Pediatrics	410	No	Negative	Weak	Sensitive	Amp, Amc, Sxt	None	-
Eco-6244	Urine	Emergency medicine	476	No	Negative	Moderate	Sensitive	Amp, Amc, Tzp, Fep, Caz, Mer, Amk, Gm, Sxt	*bla*$_{CTX-M-1}$-like	-
Eco-9744	Blood	Emergency medicine	476	No	Positive	Moderate	Sensitive	Amp, Amc	None	-
Eco-2194	Urine	Outpatient	501	No	Negative	Moderate	Sensitive	Amp, Fep, Caz, Cip	*bla*$_{CTX-M-1}$-like	Type I-E
Eco-3189	Blood	Internal medicine	501	No	Negative	Moderate	Sensitive	Amp, Amc, Tzp, Mer, Sxt	None	-
Eco-3169	Blood	Emergency medicine	569	No	Negative	High	Intermediate	Amp, Cip	*bla*$_{CTX-M-9}$-like	-

Eco-6440	Urine	Internal medicine	569	No	Negative	Moderate	Sensitive	Amp, Amc	None	Type I-F
Eco-0209	Urine	Internal medicine	646	No	Negative	Moderate	Sensitive	Amp, Amc	None	-
Eco-5740	Urine	Emergency medicine	646	No	Negative	Moderate	Intermediate	Amp, Amc	None	-
Eco-5868	Urine	Emergency medicine	646	No	Negative	Moderate	Sensitive	None	None	-
Eco-7482	Urine	Emergency medicine	646	No	Negative	High	Sensitive	Amp, Amc	None	-
Eco-2000	Swab	Internal medicine	648	No	Negative	High	Sensitive	Amp, Amc, Tzp, Fep, Caz, Mer, Cip, Sxt	None	Type I-E
Eco-2258	Urine	Urology	708	No	Negative	High	Sensitive	Amp, Amc, Tzp, Fep, Cip, Sxt	None	Type I-E
Eco-6443	Urine	Urology	744	No	Negative	Moderate	Sensitive	Amp, Amc, Cip, Sxt	None	Type I-E
Eco-0220	Urine	Internal medicine	922	No	Negative	High	Sensitive	Amp	None	-
Eco-2268	Urine	Neurology	922	No	Positive	High	Sensitive	Amp, Caz, Cip, Sxt	None	-
Eco-8704	Urine	Pediatrics	922	No	Negative	High	Sensitive	Amp, Amc	None	-
Eco-3092	Urine	Internal medicine	1011	No	Positive	Weak	Sensitive	Amp, Amc, Fep, Gm, Cip, Sxt	*bla*$_{CTX-M-9}$-like	Type I-E
Eco-9573	Urine	Internal medicine	1133	No	Negative	Moderate	Sensitive	Sxt	None	-
Eco-6378	Urine	Surgery	1195	No	Negative	Moderate	Sensitive	Amp, Amc, Tzp, Fep, Caz, Mer, Gm, Cip, Sxt	None	-

Eco-0224	Urine	Emergency medicine	1432	No	Negative	Weak	Sensitive	Amp, Amc, Cip, Sxt	None	-
Eco-5526	Pus	Urology	1538	No	Negative	High	Sensitive	Amp	None	-
Eco-3095	Urine	Urology	2371	No	Positive	Moderate	Sensitive	Amp, Amc, Fep	*bla*$_{CTX-M-9}$-like	Type I-F
Eco-6303	Urine	Internal medicine	3059	No	Negative	Moderate	Sensitive	Amp, Amc, Fep, Caz, Cip	*bla*$_{CTX-M-1}$-like, *bla*$_{CTX-M-9}$-like	-
Eco-8576	Urine	Orthopedics	3387	No	Negative	Moderate	Sensitive	None	None	-
Eco-8711	Blood	Pediatrics	3423	No	Negative	High	Sensitive	Amp, Amc	None	-
Eco-7525	Urine	Emergency medicine	3459	No	Negative	Moderate	Sensitive	None	None	-
Eco-8316	Urine	Internal medicine	4077	No	Negative	Moderate	Sensitive	Amp, Amc, Sxt	None	-
Eco-2272	Blood	Emergency medicine	4560	No	Negative	High	Sensitive	Amp, Amc, Gm, Cip, Sxt	*bla*$_{CTX-M-1}$-like, *bla*$_{CTX-M-9}$-like	-
Eco-2436	Blood	Neurology	4560	No	Negative	Moderate	Sensitive	Amp, Amc, Gm, Cip, Sxt	*bla*$_{CTX-M-1}$-like	-
Eco-2477	Urine	Emergency medicine	4560	No	Positive	Moderate	Sensitive	Amp, Amc, Fep, Caz, Gm, Cip, Sxt	*bla*$_{CTX-M-1}$-like, *bla*$_{CTX-M-9}$-like	-
Eco-2253	Urine	Internal medicine	5328	No	Negative	Moderate	Intermediate	None	None	Type I-F
Eco-2103	Urine	Emergency medicine	7399	No	Negative	High	Sensitive	Amp, Amc, Fep, Caz, Gm Cip	*bla*$_{CTX-M-1}$-like, *bla*$_{CTX-M-9}$-like	-
Eco-3088	Blood	Emergency medicine	7399	No	Negative	Moderate	Intermediate	Amp, Cip	*bla*$_{CTX-M-9}$-like	-
Eco-3205	Blood	Internal medicine	7399	No	Negative	Moderate	Sensitive	Amp, Amc, Tzp, Fep, Caz, Mer, Amk, Cip	*bla*$_{CTX-M-1}$-like	-
Eco-5568	Urine	Internal medicine	7399	No	Negative	Moderate	Sensitive	None	None	-

Eco-5835	Urine	Emergency medicine	7399	No	Negative	Moderate	Sensitive	None	None	-
Eco-6240	Pus	Emergency medicine	7399	No	Negative	Moderate	Sensitive	None	None	-
Eco-6450	Urine	Emergency medicine	7399	No	Negative	Weak	Sensitive	Amp, Cip	None	-
Eco-3104	Blood	Emergency medicine	7527	No	Negative	Moderate	Intermediate	Amp, Amc, Tzp, Fep, Caz, Amk, Gm, Cip, Sxt	$bla_{\text{CTX-M-1}}$-like	-
Eco-3113	Urine	Emergency medicine	7527	No	Negative	Moderate	Sensitive	Amp, Amc, Tzp, Fep, Caz, Mer, Cip	None	-
Eco-6060	Blood	Internal medicine	7527	No	Negative	Moderate	Sensitive	Amp, Amc, Fep, Caz, Amk, Gm, Cip, Sxt	$bla_{\text{CTX-M-1}}$-like	-
Eco-6245	Urine	Internal medicine	7527	No	Negative	Moderate	Sensitive	None	None	-
Eco-7642	Urine	Emergency medicine	7527	No	Negative	Moderate	Sensitive	Amp, Amc, Tzp, Cip	None	-
Eco-3281	Blood	Emergency medicine	9312	No	Negative	Weak	Sensitive	Amp, Amc, Tzp, Fep, Caz, Mer, Amk, Sxt	$bla_{\text{CTX-M-1}}$-like	Type I-E
Eco-3142	Blood	Surgery	9612	No	Negative	High	Sensitive	Amp, Amc, Tzp, Fep, Caz, Mer, Amk, Gm, Sxt	None	-
Eco-6324	Urine	Internal medicine	9612	No	Negative	Moderate	Sensitive	Amp, Amc	None	-
Eco-6329	Urine	Emergency medicine	9612	No	Negative	Moderate	Sensitive	None	None	-
Eco-7862	Blood	Emergency medicine	9612	No	Negative	Weak	Sensitive	Amp, Amc, Sxt	None	-

Eco-3116	Urine	Internal medicine	10605	No	Negative	Moderate	Sensitive	Amp, Amc, Tzp, Fep, Caz, Mer, Cip, Sxt	*bla*$_{CTX-M-1}$-like	-
Eco-7522	Blood	Emergency medicine	10605	No	Negative	Moderate	Sensitive	Amp, Amc, Tzp	None	-

Table S2. Sequences of spacers characterized from *E. coli* isolates of Greek origin.

SPACER	CRISPR/Cas type	Spacer name	Origin
ACATGAATGTCGGTTCAGACCGTGTTTTTACC	IE	E1	plasmid (DNA-binding protein CP041576.1)
TCGTCGGCGTCTTCACCCGTCGCATAACGCTG	IE	E2	chromosome (-)
TGCCAGCGTGTCGGCGTGCGTTTCGCGCTGCC	IE	E3	chromosome (-)
GTTGCTGCTGCGTTCGATATTATTTTATACCC	IE	E4	chromosome (-)
TCGATCGATAGCTGCGCGATACCTGCGGGGAT	IE	E5	chromosome (-)
GGACTCGCCGCAGGTGAGGAAAACGCAATAAC	IE	E6	chromosome (-)
GTTATTGCTGCCTCCGTCACCTGTCCGATCGG	IE	E7	chromosome (-)
AATTTCTTCAGCCAACTTTTTTCGGACCGTCA	IE	E8	CRISPR (OR645744.1)
CGCCCGTTTGAACAAAAACGGGTATCTAACTG	IE	E9	chromosome (-)
ATAATTGATCGTGATTATCACGTTAGTGCCGC	IE	E10	chromosome (-)
CTGTTGGCAAGCCAGGATCTGAACAATACCGT	IE	E11	plasmid pB27_2 DNA (hyp protein AP027426.1) Bacteriophage sp. isolate 3121_76502, (hyp protein OP075223.1)
CAATTTGGCAATCGGGGCGACTATCTTTGGAT	IE	E12	chromosome (-)
TTCTCGTTACTGGCGACCAGTCGAGGCGCTGG	IE	E13	chromosome (-)
GGCAGCTATCGAGGCAGTTATCGAGCGTCGCG	IE	E14	chromosome (-)
CAAAGGACACCGGGAGGCACCCGGCACCGCAG	IE	E15	plasmid pUSDA5905_1 (CP039843.1), Bacteriophage sp. isolate 3509_17389 OP075566.1 (hyp protein), Escherichia phage vB_EcoM-813R1 (ON470617.1)
GGTGAGAATCTGAATTATTAATTCCAGTTCGT	IE	E16	chromosome (-)
AATGCATCAGTTGAACACAAAAGTASCTTTTC	IE	E17	chromosome
ACGGATTGCGGTATATGCAGTGTCCAGATTCT	IE	E19	chromosome
AGTGCTGGACCATTTCAAATTCACAATAGATT	IE	E20	chromosome
CTGTCCCAGGCCGAGGCTGTATTTCAATCCTG	IE	E21	chromosome
GCGATGGATCAGGGTATTACACTCGACGGCGG	IE	E22	chromosome
GCTGCGGGTCAATCAACTGGAGTGCAAATCCC	IE	E23	chromosome
GGAATGATATTTCAATAAATAATTATAACAAT	IE	E24	chromosome
GGGGATAACGGTTATCCACTGGCCGCCGATCT	IE	E25	chromosome

GTCGCGCGCCGACGGAATAAACCGAATAAATT	IE	E26	chromosome
TAAACCACCAGCCAGACCACCAATTACCACAC	IE	E27	chromosome
TCGTCAGGGCACAATAACTGAGGTTCGCACAA	IE	E28	chromosome
TGAGCGTCGGCGGCTCGCTGGATTTGCGCGG	IE	E29	chromosome
TGTAATAGCCTGATCTCTGATCTCCCTCGCCT	IE	E30	chromosome
TTTTGCTGACMCCGGCAATACTGAACGGCTGG	IE	E31	plasmid unnamed(DNA cytosine methyltransferase CP122665.1)
TTTTTGTTAATTGCGTTTGCCACGGTTTGCAA	IE	E32	plasmid p24C171-1 (LC501671.1)
GCCGGAAAATATTCATGATGGGGGTGGTTATGG	IE	E33	CRISPR (JF495856.1)
GGGGCCGCCGTGTTCACATTTGCCTAATTTCC	IE	E34	CRISPR (JF495856.1)
AACATGTCTTTCGTCAATTTTGGCGCTATAAA	IE	E35	CRISPR (JF495856.1)
TATATAAACGAGGTGAATTTATGGAAACTGTA	IE	E36	CRISPR (GU260803.1)
GTTAGTTTTCTGAAAAAATTGTTTTCTTACGC	IE	E37	CRISPR (JF495856.1)
AATATTGTCGTGGACAAATGCCGGTGTTATTG	IE	E38	CRISPR (JF495856.1)
TTCGAGACACTTCTCTGCAAAATTAATGAAAT	IE	E39	CRISPR (JF495856.1)
CAGGTATCAAGAAATAATCGAAGCCTTCATGA	IE	E40	CRISPR (JF495856.1)
TACCGTACAGACTGCCGGATATTATTTTTTTG	IE	E42	CRISPR (CP158329.1)
CGACGTTCGACGAAATGCGCGATGGCGGTCGG	IE	E43	CRISPR (JF495856.1)
TTCTGCGTCCCCGATGTCTGGCCCGACTGGTC	IE	E44	CRISPR (JF495856.1)
TATATGACCGGAACACTTCCGCCTGACGTTTG	IE	E45	CRISPR (JF495856.1)
GTCCTGTTTTTGACCGGCGGTAAATGTTGCCTG	IE	E46	CRISPR (JF496009.1)
AATATCCGTTCCTCGCTCTGCGTTTTCGTTGA	IE	E47	CRISPR (JF496009.1)
GGTCGGGTATTTCATTATGCATCCTGAACTAA	IE	E48	CRISPR (JF496009.1)
TATGCGCTTGCTGCTGCGTTGATGTACGCGGC	IE	E49	CRISPR (JF496009.1)
TTAACTACCTGAACACCGGGAAGACTCCGGAC	IE	E50	CRISPR (JF496009.1)
GATTCAATGTCCGACGCATCCAAGTAAATATT	IE	E51	CRISPR (JF496009.1)
GGATGAGCAGGGAGCAACAAAAGTAGCCGGAA	IE	E52	chromosome (CP028771.1)
CCATACATTAACACGGTGTGGCCACATTTCAA	IE	E53	CRISPR (JF496009.1)
GCGCGGCCGCGATATCGTTAGCACTGGCTCCCG	IE	E54	CRISPR (JF496009.1)
CAAAACACCTGAACGTTGAGATAGGGCTAAAT	IE	E55	CRISPR (JF496009.1)
GTTGTCGTATTCCTTAGAAATCTGATGCCAGCC	IE	E56	CRISPR (JF496009.1)

TGACTGACCGGGAAAAGAAAACACCTGGCGAA	IE	E57	CRISPR (JF496009.1)
AATTTTTCCAGTGGTGTCGTTGTAATTAAGGA	IE	E58	CRISPR (JF496009.1)
AGGTTAAAGAGGCGCGCCGGGCGCTGGCTGGG	IE	E59	CRISPR (JF496012.1)
AAGAGAACAGCTTATATCTGATGCCGGTTTGC	IE	E60	CRISPR (JF496009.1)
ACACCGGCGAGATCGTATTTTTTGCCGCTGAG	IE	E61	CRISPR (JF496009.1)
TCAGTGATATCCATCATCGCATCCAGTGCGCCC	IE	E62	CRISPR(AP023190.1)
CAGCGTCAGGCGTGAAATCTCACCGTCGTTGCC	IE	E63	CRISPR (CP076470.1)
TCGGTTCAGGCGTTGCAAACCTGGCTACCGGGC	IE	E64	CRISPR (CP076470.1)
GTAGTCCATCATTCCACCTATGTCTGAACTCCC	IE	E65	CRISPR (CP076470.1)
AGCAACGATAACCACAGAATACGGAGAACTGGT	IE	E66	CRISPR (OQ174873.1)
GACCGCCGACAGATTGCGAGTACGTCTGGCTAC	IE	E67	CRISPR(AP023190.1)
AAGCCCGTGGCGCGGGTCGCCGCTGGCGTCAAC	IE	E68	CRISPR (CP082827.1)
AGCCCGCCTGGTCATCTCAACGCAGCATTACTC	IE	E69	CRISPR (OQ174892.1)
TTTACGCAGCCATCACCGAAATTAACTATTCCC	IE	E70	CRISPR(AP023190.1)
GGCCGCTGAACGTTTCCCGTCGCACCGGTGTGC	IE	E71	CRISPR(AP023190.1, OQ174892.1)
CGCGCACCGGTACTGAGGCACAACAACGAGAGC	IE	E72	CRISPR (OQ174892.1)
CCGTGCCATTTTTTGCGCCGCCGAGCCTTTATC	IE	E73	CRISPR (OQ174892.1)
TTATTGATATCAGCGATGAGGAGGTTTATCGCC	IE	E74	CRISPR(AP023190.1) chromosome (DnaB-like helicase C-terminal domain-containing protein CP128448.1)
TATTTATTGTGCAATTAGTTTTGCATTAAATGC	IE	E75	CRISPR(AP023190.1)
GAGCCCTGCCAGAATGGGGCCTCTTTTGTACC	IE	E76	CRISPR(AP023190.1)
CAGATGACCCGCGTTGTTACGAACAAACTCAA	IE	E77	CRISPR (CP055624.1)
TGGTGATCCCGCAATCCACGACAAAATACTGG	IE	E78	CRISPR(AP023190.1)
GATCGCCTGCTCCAGCGTCACTCCCTGCCCCT	IE	E80	CRISPR(AP023190.1)
TAATCCGATATCAGTGGGCGCCGCTTTACTGA	IE	E81	chromosome(lysozyme inhibitor LprI family protein CP103562.1) CRISPR(AP023190.1)
ACACCGCAAGGCCTGACGAGAAATCGCCAGGC	IE	E82	CRISPR (JF496170.1)
TGCCTCGGCGGCGGCTCTGCGCAACCGCGAAA	IE	E84	CRISPR (CP082827.1)
GTTAAACCGTGATGATCAGCAAAAGCTATTTC	IE	E85	CRISPR (CP082827.1)
GTCAATAGGCGGCGTCCCGTAGCCGTCCCCTT	IE	E86	CRISPR (CP082827.1)

TTGAAATGGTCCAGCACTGCGACCGTCGGCCC	IE	E87	CRISPR (CP082827.1)
GCTGGTGGCGCGGGCAAACGGAACAATCCCGC	IE	E88	plasmid pSvP1_F (DarB MN510446.1)p28Eco12 (helicase CP038506.1) UNNAMED (N-6 DNA methylase CP099043.1) bacteriophage sp. isolate 2015_6367 (MAG: SNF2 family OP076414.1) Escherichia phage vB_EcoM-813R1 (defense against restriction protein A ON470617.1)
ACCAAGAAGCTGACGAATCAGCCCGACACGCA	IE	E89	plasmid unnamed(CP141089.1)
CCAGTTTCATATGAGCCGCCGCGCTGGATCAT	IE	E90	CRISPR (CP134389.1)
ACAAATGATGCGCCAAAACCAAGACTTTTACA	IE	E91	CRISPR (JF496106.1)
TGATCAATCTGGTTTATATAGGCTTGCGCGGA	IE	E92	CRISPR (CP082827.1)
CTGCAACTGATTTTACGCGAGCGCAGGCCGCG	IE	E93	CRISPR (CP134389.1)
AGTAATCTCCGATCTGTTTGTAAATCTGCTGC	IE	E94	CRISPR (CP134389.1)
GAGGTCGCAGTCCTGCTCAATCTGCCCCGTGT	IE	E95	CRISPR (CP134389.1)
CAAATCACAAATTTTCGCAAAAGCCTGGGCAA	IE	E96	CRISPR (CP134389.1)
ATCGGGGTATTCAGTTTCGATGCGGGATTGTA	IE	E97	CRISPR (CP134389.1)
ACCATCACCAGGCACTACCGGCACTGGCTGCT	IE	E98	CRISPR (GU260885.1)
TCCGGGCCATCCATACACCTTCTTAACCCGGC	IE	E99	CRISPR (OR645716.1)
CCCCACGCATTGTACTCTGTGCAGGCGTAACC	IE	E100	CRISPR (OQ174968.1)
AGCCGGATGCTGATCGATACCGCGCATCACCG	IE	E101	CRISPR (OQ174859.1)
AAAACCACCGGACCTGACCATTTGAAATGAGC	IE	E102	CRISPR (OQ174968.1)
GCTGCGCTCACCGTCGCCAGCTTTTTCCAGTA	IE	E103	CRISPR (OQ174888.1)
CGGTCAGGGGAACAGCATAACAATTCCGTGCC	IE	E104	CRISPR (OR645730.1)
CGGAACTGGTCCAGCGTTAACCGGCGGCGGAT	IE	E105	CRISPR (OQ174888.1)
TGTTGCAGCGGCGCACCGCTGACGGACTCGCC	IE	E106	CRISPR (OQ174913.1)
ATGTAATGAATATTAAGCTTTTCACATTGTCC	IE	E107	CRISPR (OQ174952.1)
CTTTATTCCATCCCTTAATGCTTTGATGAAAT	IE	E109	CRISPR (OQ174913.1)
ATAAGCCCCGGCATCAGTTGCAGCCGCGGACG	IE	E110	CRISPR (OQ174852.1)
TCATCATGACGCACGGGCGACCCGACAAGGTT	IE	E111	CRISPR (OQ174852.1)
GAACGCGAGATGCACCCAGGTTTGTGGGAACT	IE	E112	CRISPR (OQ174852.1)
GCGGTCGATTTTGCTGGCGTTGTTTCGGCTGG	IE	E114	CRISPR (OQ174872.1)
CCCCGCTTCGCTCGAGTACCCGCTCTATATCC	IE	E115	CRISPR (OQ174852.1)

GACTCAATGGCAACCGTCACGGCCTGTCCTGG	IE	E116	CRISPR (OQ174852.1)
TTGGTTGTTCGGCGTTTTGAAGAGAACAAAAA	IE	E117	CRISPR (OQ174852.1)
CAAGTGATATCCATCATCGCATCCAGTGCGTC	IE	E118	CRISPR (OQ174877.1)
ATCACCAATGAGGGTGCATTCCGTTGGCACGC	IE	E119	CRISPR (GU260849.1)
GAAATGCTGGTGAGCGTTAATGCCGCAAACACA	IE	E120	CRISPR (CP076470.1)
CTGTTTTCGCAAATCTATGGACTATTGCTATT	IE	E121	CRISPR (CP060867.1)
GACAGAACGGCCTCAGTAGTCTCGTCAGGCTC	IE	E122	CRISPR (CP060867.1)
ACGCGCGTACCGGATCGCGGACAACAAATTGC	IE	E123	CRISPR (KF707526.1)
TTGCTGAAAAAGAAGGCTCCGGCGTTATCAGT	IE	E124	CRISPR (KF707526.1)
TAGTTAACTTTTAGACAGAATATCCGTGTACC	IE	E125	CRISPR (KC765644.1)
GGTTTTACAAGCTTAAATGATTTTAAATTTTG	IE	E126	CRISPR (KC765644.1)
AACTGGATTCTGCTCTAATTATTACATCATCG	IE	E127	CRISPR (KC765644.1)
GGAAACCGCCCCGGCGGTGGTTAGCTGTTTGC	IE	E128	CRISPR (KC765644.1)
TTTGCTACCCGCTCAAAACCGCCGATTCGCGT	IE	E129	CRISPR (KC765644.1)
TCGGCCAGCGTCCAGGCTGGCACCCGATATTC	IE	E130	CRISPR (OQ174970.1)
CTGCCGACTGACCGCCGACGCTTTCGCGTTAA	IE	E131	CRISPR (OQ174970.1)
GGAATATCGTTGCGCTAAAAGAGATTCTGGGC	IE	E132	CRISPR (OQ174970.1)
CCTGGTGTTGTCTGTTTCGTGACGCTCGGTAA	IE	E133	CRISPR (OQ174970.1)
CTATAATTTGTTTTTGCACCACTGTACCTCGT	IE	E134	CRISPR (OQ174970.1)
TCTTCGGGCGCGCGTTTTCGCAGACCAGCGAT	IE	E135	CRISPR (OQ174970.1)
GTTACGCTGCCTGAATATCGCAATTCCCCTGA	IE	E136	CRISPR (OQ174970.1)
TGCATATCAGATGGGCACTGCTGATTGGAGAA	IE	E137	CRISPR (OQ174970.1)
ATTAAATTTTGCAGGTAGCTGTTATCCGTGTC	IE	E138	CRISPR (OQ174970.1)
CGATTCCTCGTAGCTGCCACTCGACTGAAAGC	IE	E139	CRISPR (OQ174970.1)
GTCGCCTGTTCAACTTTAAACGGCTCAAACCA	IE	E140	CRISPR (GU260808.1)
TAATCACGTTTTAGCGCGCCCTCGTCCGGTTT	IE	E141	CRISPR (KC765367.1)
ATCACGATAACGCTGCTGTGATTCGTCCCCGT	IE	E142	CRISPR (KC765367.1)
AAGACGACGTGATCCGCAAAGTCGAAGGCACG	IE	E143	CRISPR (KC765367.1)
TGTTTTTCTGCCGGTTTAACACGGTGCGATAA	IE	E144	CRISPR (KC765326.1)
TGTTTCTGTCCCAACTGAACAGCGCCGATAGT	IE	E145	CRISPR (KC765367.1)

TTGTCCAGACTCCCGATTGTTTTGCCGTCCGC	IE	E146	CRISPR (CP173484.1)
CTGGTTTCTTTTTTCTGCTCCGGTGAGAGTT	IE	E147	No significant similarity found
GGACTTAAAGATCGCATCACAGTACTGGAGGG	IE	E148	CRISPR (KC765326.1)
GGCAATCCGTTGGATTCATTGTTTTGTTGCTC	IE	E149	CRISPR (JF496055.1)
AATAACTCGCGTAAATGCTCTGCGGCGCTACG	IE	E150	CRISPR (JF496055.1)
CCTCCTGTACCACGATCGCGCCCTCATACCCC	IE	E151	CRISPR (JF496055.1)
GTCCCCGATCGGAATGGCGACATGCGATGCCT	IE	E152	CRISPR (JF496055.1)
CAAAAAACGCCGCGTTTTTTGCATTTTATCGG	IE	E153	CRISPR (JF496055.1)
CCCAGGCCAAGCATTATATCCTGTGCGTCGTT	IE	E156	CRISPR (CP134389.1)
TTCTGCCGCGCCAGCAAACGTATCGGCGGTCA	IE	E157	CRISPR (CP134389.1)
GTATTTTTTATCGTCGCATGAGTCGCGGTAAC	IE	E158	CRISPR (CP134389.1)
TTGCGTTATTTCTATCTGGTGAGTCGCGGCAC	IE	E159	CRISPR (CP134389.1)
AGAAATCAAACTCAGCGTGCAAATGGGGTAGC	IE	E160	CRISPR (baseplate J/gp47 family protein CP068806.1)
CACCCCTGATATTTTTGCCACCGTGGTTACTC	IE	E161	CRISPR (CP134389.1)
CTGGATTTCACCTCAGCAAATGCTGGATGTGG	IE	E162	CRISPR (CP134389.1)
GGCCATTCCCCCAAGCAGACGCAGGAAGTTAA	IE	E163	CRISPR (CP134389.1)
GCTAACCTATAACACGGAAAATCACACGCTAA	IE	E164	CRISPR (CP134389.1)
TCCGGTACGCGCGGGATCGAATCCGGCCCAGA	IE	E171	CRISPR (CP134389.1)
TTTCCCCCATGCGGGAAGCGCGTGCGATTTCG	IE	E178	CRISPR (CP134389.1)
CAACGAAATTGAGAGCAAAGGTCGCTACGCAG	IE	E180	CRISPR (OZ038578.1)
TTCATGAAGCACGCTGGAATTTATATTTGCCC	IE	E181	CRISPR (KJ500244.1)
GCGCAGCCCGATGCGGTTAGAGATATAGGCCTC	IE	E182	CRISPR (KJ500244.1)
TTTACGTCGAAGCGCCTTTTTCGCCTCCAGGT	IE	E183	CRISPR (KJ500244.1)
AAAAAATGATGCTGTATTTCACTATTGGGTTC	IE	E184	CRISPR (KJ500244.1)
CCGTTAAAATATTCGCCCGTTGTGTAAGCGTA	IE	E185	CRISPR (KJ500244.1)
ATCTCCAGCGCCTTTTCGACGCAACGGTGCGC	IE	E186	CRISPR (KJ500244.1)
TGACCGATTTTATTGATCGCCCGATAGACCTG	IE	E187	chromosome (phage tail sheath subtilisin-like domain-containing protein CP102061.1)
CCAGATTCCGCTTTCTCTACCTTCTCTTTTTC	IE	E188	CRISPR (KJ500244.1)
GGGTTGTGCGCCCTGGTGGGCGAAAACGGTGG	IE	E189	CRISPR (KJ500244.1)

ATTTTCCTGATCGAGTTTTGAGAATTTTGTCG	IE	E190	CRISPR (JF495822.1)
TTTTGTGCCCTGGCGTTTTGCTGGCTCTCGCG	IE	E191	CRISPR (KJ500276.1)
TTTGCCGCTGTCAGCATTGCTGGCGCGTAATA	IE	E192	CRISPR (KJ500276.1)
AAAATTCATATTGATAAACACCGCGTTTGTAT	IE	E193	CRISPR (KJ500276.1)
TTTACTACAATAGGTGGACACGCTACGGCGCA	IE	E194	CRISPR (KJ500276.1)
AAATATGGGGAGCGCCGTTAATGAAATCAAAA	IE	E195	CRISPR (CP049343.1)
CGCCGCGGACTCACGAGCAGGCCATCACAGAC	IE	E196	CRISPR (KJ500276.1)
CAGTTGTCTGATGTGTCGCTGGCCTGGCGGCA	IE	E197	CRISPR (KJ500276.1)
CCGTTCATATTCGTTTCCTCGTGGCGCGATCT	IE	E198	CRISPR (CP049343.1)
GGACCAAATACCCGTCCCTCAATCCACGCCGT	IE	E199	CRISPR (CP049343.1)
AATTGAGACGTTCCACGCAATTAAACAAGCCG	IE	E41	CRISPR (CP125923.1)
TTGTTGATAAAGTGGCGCGCCAGGTGCGTTTA	IE	E201	CRISPR (CP125923.1)
CGGTCGTCAGGTACTAATCTAAAGGCTGTTTT	IE	E202	CRISPR (KJ500245.1)
GTGGCGTCCGTCGTCGTTTAAAGCGTCAATGC	IE	E203	CRISPR (CP125923.1)
ACGTCAACCTCTTTGAGGCTGGCTGTGATGAT	IE	E230	chromosome (type I restriction enzyme HsdR N-terminal domain-containing protein CP057104.1)
CCTTTTGATATTCGTGAACCAGACTTTTAGTA	IE	E231	CRISPR (OQ174852.1)
AGGCTCAATAATGCCGCCGCGAACTCCAACGC	IE	E232	CRISPR (OQ174895.1)
TTCATTATAAAAATCCATTTTGATTAAAGACT	IE	E233	CRISPR (KF523179.1)
CATTAATTGGGAACGCCTCATCACTGCCACCG	IE	E236	CRISPR (OQ174951.1)
AATTCATAAATTAATTTACTATTTTGAGTGCG	IE	E237	CRISPR (JF495833.1)
GTTAGGCAGGTGGCGATGTTCAGCGCCGGATG	IE	E238	CRISPR (OQ174878.1)
CAATACGACCGCGAATAAACATGATGTGATCA	IE	E241	CRISPR (KF523179.1)
AAACAGATTGTTCGTTTTCCCCATATTCATGA	IE	E247	CRISPR (KF523179.1)
TAATCTCGTTTGACGATTTCATTGCTGAAGAG	IE	E253	CRISPR (KX880986.1)
TAGAAATGACTAAAAACGCGAGGCGGCGTAAT	IE	E254	CRISPR (CP088131.1)
CCCCGGAGACTGGCGCAACAATCCCGACCACG	IE	E255	CRISPR (KX880985.1)
AGAGCAACAGGTATTTCAAATGGCCAGCCGTG	IE	E256	CRISPR (KX880986.1)
TTCAGCAATCTGAAACCAGTTGGCTCGCTGTT	IE	E257	CRISPR (KX880986.1)
GCCCGCCTCGTCGGTGTATTCCGCGAGATCGC	IE	E258	CRISPR (KX880986.1)

GGCATAGCCAGGCTGATCCGGCGACGGCCTTA	IE	E259	CRISPR (KC765335.1) plasmid (OL790696.1)
TACGTGAAGAATATTTGCAACACCCGCAAGAA	IE	E260	CRISPR (KX880985.1)
AACATCGGAAACGGCTTCGCGGCGGCGGCGTC	IE	E262	CRISPR (KC765335.1)
AGTGCTGGACCATTTCAAATTCACAATAGATT	IE	E264	CRISPR (CP056821.1)
TGATATCCTGTCCAATACTGACGCGGTTATCG	IE	E265	CRISPR (CP122507.1)
TAATACATGGGGCTACTGGATTATTATTTATT	IE	E266	CRISPR (CP122507.1)
CGTCCGGATCGGTTTCGAGAATCTCTACGCTC	IE	E267	CRISPR (CP122507.1)
CTGTTTTCGCAAATCTATGGACTATTGCTATTC	IE	E268	CRISPR (CP142438.1)
GGGCGCACGGAATACAAAGCCGTGTATCTGCTC	IE	E269	CRISPR (CP142438.1)
TGGCTCTGCAACAGCAGCACCCATGACCACGTC	IE	E270	CRISPR (CP142438.1)
ATTACGCCTTTTTGCGATTGCCCGGTTTTTGCC	IE	E272	CRISPR (CP142438.1)
GCGTGTCGGCAAATCCCGAGGAAATAGAACCCC	IE	E273	CRISPR (CP142035.1)
CGAATCACGCCCCGCTTTTTACCGCGTCAGCGC	IE	E274	CRISPR (CP147084.1)
CTGTGCTCCTGTTTTGTGCCGTAGTTACCGATC	IE	E275	CRISPR (CP122507.1)
ACGTTCGCACCGGTCAGGGTACTGCGCAGCGT	IF	F1	CRISPR (OZ039952.1)
CTGAACGTTGAAGAGTGCGACCGTCTCTCCTT	IF	F2	plasmid unnamed (antirestriction protein CP098942.1)
GAAACCAGAGCGCCCGCATAAAACAGGCACAA	IF	F3	CRISPR (CP165590.1)
GAATATTTTGGAAAAATAGCTATCAATCCGGG	IF	F4	CRISPR (CP165590.1)
GATACGATCGAATTTAGTCACTGAATACCCCC	IF	F5	CRISPR (CP165590.1)
GCGCGCAGTGCCTGATAATCAATTTTGCTCAT	IF	F6	CRISPR (OZ039952.1)
GGGTTGCGCAATGGGCTGGCCGACGAACGCGG	IF	F7	plasmid pAVS0973-C (CP124474.1)
TGACGCCATATGCAGATCATTGAGGCGAAACC	IF	F8	CRISPR (CP165590.1)
TGATGGCGCAGCAGTCCTCCCTCCTGCCGCCA	IF	F9	plasmid pAVS0973-C (CP124474.1)
TGGATTCCAAACCGCCCACCAACAAAAACAGGT	IF	F10	CRISPR (OZ039140.1)
TGTGGCGCTGATGCGTCTGGGCGTCTTTGTAC	IF	F11	plasmid unnamed (RepB family plasmid replication initiator CP098932.1) pOXA-484_IncF (RepFIB replication protein A OP594535.1)
AGCACGGCTGCGGGGAATGGCTCAATCTCTGC	IF	F14	CRISPR (OZ039140.1)
TCAGCCGGAGGCTCTCAATTTCAGCCGCGCGG	IF	F15	CRISPR (CP165590.1)
GCCAGCATAAAACCGCCTTTGATATTTTATTG	IF	F16	CRISPR (CP165590.1)

TACACAACAGGCGAATATTTTAACGGCGAAAC	IF	F26	CRISPR (prophage tail fiber N-terminal domain-containing protein CP172127.1)
AATATCCGCCAGCGGGCTTACTGACGGCATCA	IF	F27	CRISPR (OZ038578.1)
GGTACTGCGGTTTCTGTTGTTGCTGGCTCAGC	IF	F28	CRISPR (OQ174883.1)
GTTAACCGTTTCACACTTAACACTATGAACCG	IF	F29	chromosome (GPO family capsid scaffolding protein CP056928.1)
AAGCGGCTCCGGCCGCTTTTTTTCGGAATTTAT	IF	F30	CRISPR (OZ038578.1)
AGCAGCGGTGGCGAACCGCGCGTTGTTGATTT	IF	F31	CRISPR (OZ038578.1)
AGAACTCGAAATTTATCGCGGGACTTTTCAGC	IF	F32	CRISPR (OZ038578.1)
AGGCTGCTGGCGCTCTCGTTGTTAACGGCAGC	IF	F33	CRISPR (OZ038578.1)
GTTCCACGCGGATATCTGTAATTTCCAGCAAA	IF	F34	CRISPR (OZ038578.1)
CAGAACAACTATCACTTCGGAGGGAGCACGCTG	IF	F36	plasmid (IncF plasmid conjugative transfer pilus assembly protein TraH OQ423473.1)
GGTAGGACTCGTTGCGCCAGCGCTTGTATTCG	IF	F37	CRISPR (OZ038578.1)
GTCAAATAACAAAGCCCACTTACCGGGGGCGC	IF	F38	CRISPR (OZ038578.1)
GGTTGCAACCGCCGGCGCGCCCAACCGCCCTC	IF	F39	CRISPR (OZ038578.1)
AACACGCTGGACTCGTACCGGCTCAACTACTA	IF	F40	CRISPR (JF495988.1)
CGCCCCGGTCACATTGATTGTCTGATACACGT	IF	F41	CRISPR (OZ038578.1)
GATCTCATTGTTGGTCTGTGGGGTGGTCTGGA	IF	F48	chromosome (CP042250.1)
TGGAAGGTGCATCAAAAGTTGCAGCAGAATCGA	IF	F54	plasmid (type IV secretion system protein CP164402.1)
AGTGCACCATTTCTTGTGAGTTAGCGATTGCAT	IF	F55	plasmid (type IV secretion system protein CP172162.1)
GGAAAGCAGCCGAAGCCAAAGGTGATGCCGAA	IF	F56	CRISPR (OR645713.1)
GGAAAAATTCACCCGTTCGTTGTAAAAAATGG	IF	F57	chromosome (CP055753.1)
ATCAGAAACACGGTATCCAATCATCATCTCAG	IF	F58	No significant similarity found
GCAGAACCCGCAGACATAAGACCCGCTTTTGC	IF	F59	No significant similarity found
TTAGCCCTGCCTTTCCTGATTGTTTCGCGGTA	IF	F60	chromosome (CP091029.1)
GTCACGCAACATGCGGTTGCAAATGAATTTTT	IF	F61	chromosome (CP091029.1)
CGTCAGGTAGCTCTAAACAATACCAGGCCCGA	IF	F62	No significant similarity found
GAATTCGCGAGAAAGGCCTGAATGTAGAGGGG	IF	F63	No significant similarity found
TGGCGGAGTGGGGGCAGGTTCAGCAACCGTAG	IF	F64	No significant similarity found
AGCAATAGCATTAAGCTTTCACTGATGAGTTA	IF	F65	No significant similarity found

AGCCCGATTTATGCTCGCCGCCGAACGATACG	IF	F66	No significant similarity found
AGACACTCTCTGGTGGTGAGAAGTGGAAGGTT	IF	F67	plasmid (AAA family ATPase CP137164.1) (SMC family ATPase CP122519.1) (exonuclease AP027572.1) (93.75%)
TGTTACCAAATGACAGATGCATCTTTGGAGAT	IF	F68	plasmid (CP038361.1)
AGCCTGAGTCGAGGATATCCCTGCTCCTGGAA	IF	F69	plasmid (transcription termination/antitermination NusG family protein CP163994.1)
AGCACTCGTGTAGCCCAGCAATAACAGCGGAA	IF	F70	chromosome (CP058207.1)
TGAAGCTTCGCAAACCTCTCTCCGCAGATACC	IF	F71	plasmid (type IV secretory pathway protease TraF CP042584.1) (S26 family signal peptidase CP057715.1)
ACCGCCGGGGCGGCTTCCCCGATAAAAAGGAT	IF	F72	CRISPR (OZ038955.1) (97%)
AGTACTCGATTTGCAAATCAGCGTTATCGTCC	IF	F73	chromosome (CP055753.1)
CAGCAACACAGCAGTTGACGCGAATGCCCCCG	IF	F74	No significant similarity found
GGAGAACGGCGGGAACGCGGTAGCACCAATGAT	IF	F75	No significant similarity found
GTTGATTGCGGGATTCGTTTTCCGCAGATAAC	IF	F76	No significant similarity found
TTGAGCTGGATTACAACGTCCCTGCAATTGGC	IF	F77	plasmid (CP137164.1)
GAGATTAACCCGTATGATTGCGCTGTCGAGTT	IF	F78	No significant similarity found
AGTACGTTACGGGTCAGACTGATGGTTTCATT	IF	F79	plasmid (conjugal transfer protein TrbC CP042584.1)
CATGGAGCGGCGTAACCGTCGCACCGGCAGGA	IF	F80	plasmid (Tyrosine recombinase XerD PP320254.1) (site-specific integrase CP145945.1) (resolvase AP026102.1)
GCCAACGCGGAGATACGCCCCGGCTGCGGGTG	IF	F81	chromosome (CP057804.1)
AACACCATAATAAGATTCGCCGTTTATAGTAA	IF	F82	CRISPR (OZ038955.1)
GCTAGGGGATGGATTGTTTGAGATACGAGCAA	IF	F83	CRISPR (CP138217.1)
AAATGCGGCCAGCATATTGCGCTGCTCGATCG	IF	F84	chromosome (OZ038955.1)
ATGACAAAACTGATATCTGACTCCAGATATTC	IF	F85	CRISPR (CP138217.1)
GGTAAGTGGGTTTTGTTATTTGACGGGGGAAT	IF	F86	CRISPR (CP138217.1)
AGGTTGAGGCGACGGCAACTAAACTGCGTGGC	IF	F87	CRISPR (OZ038955.1)
ACGTTGTAGCAAAACACTGCCGGGCCGTGACT	IF	F88	CRISPR (OZ038955.1)
AGACTGCGGTTTCAATGCGGCGGATATTTTCGA	IF	F89	CRISPR (CP138217.1)
AGCGAGGCTGCATACGTTGAGTGCCCACACTG	IF	F90	CRISPR (CP138217.1)
GCATGAGGCACCGCCCTGCACCGGTATTTTAT	IF	F91	CRISPR (CP138217.1)

ACATTGCAATTAACTGATATCGCACCATCATCGG	IF	F92	CRISPR (OZ038955.1)
TACATACTCAAACGATTACTGAATCACAAAAC	IF	F93	CRISPR (CP122507.1)
CGGCTGCACGAATTCGCGAGCGAACTGTTTCT	IF	F94	CRISPR (OZ038955.1)
GCCCGTTCATGCGCTTCATTAGCGCGCGTTCG	IF	F95	CRISPR (CP138217.1)
GCAAAGTGGGCGCTATTTCATATCCTGCATCC	IF	F96	CRISPR (CP138217.1)
TTTTAATTTTTCCAGATAATTTTGGCGAGTCT	IF	F97	CRISPR (CP138217.1)
AGGAGCATTGGCGGAGGGAACGCGAGCGCGGGT	IF	F98	CRISPR (JF495984.1)
GAATCTCGGCGCGACGGCACCCGCCACCGGGG	IF	F99	CRISPR (JF495984.1)
CAGCGGGGTGATTGAATATTTTGGCATCAGAC	IF	F100	CRISPR (JF495984.1)
TTTCTGCTGTTATCGCCGGGTTGCACGAGTGC	IF	F101	CRISPR (CP138217.1)

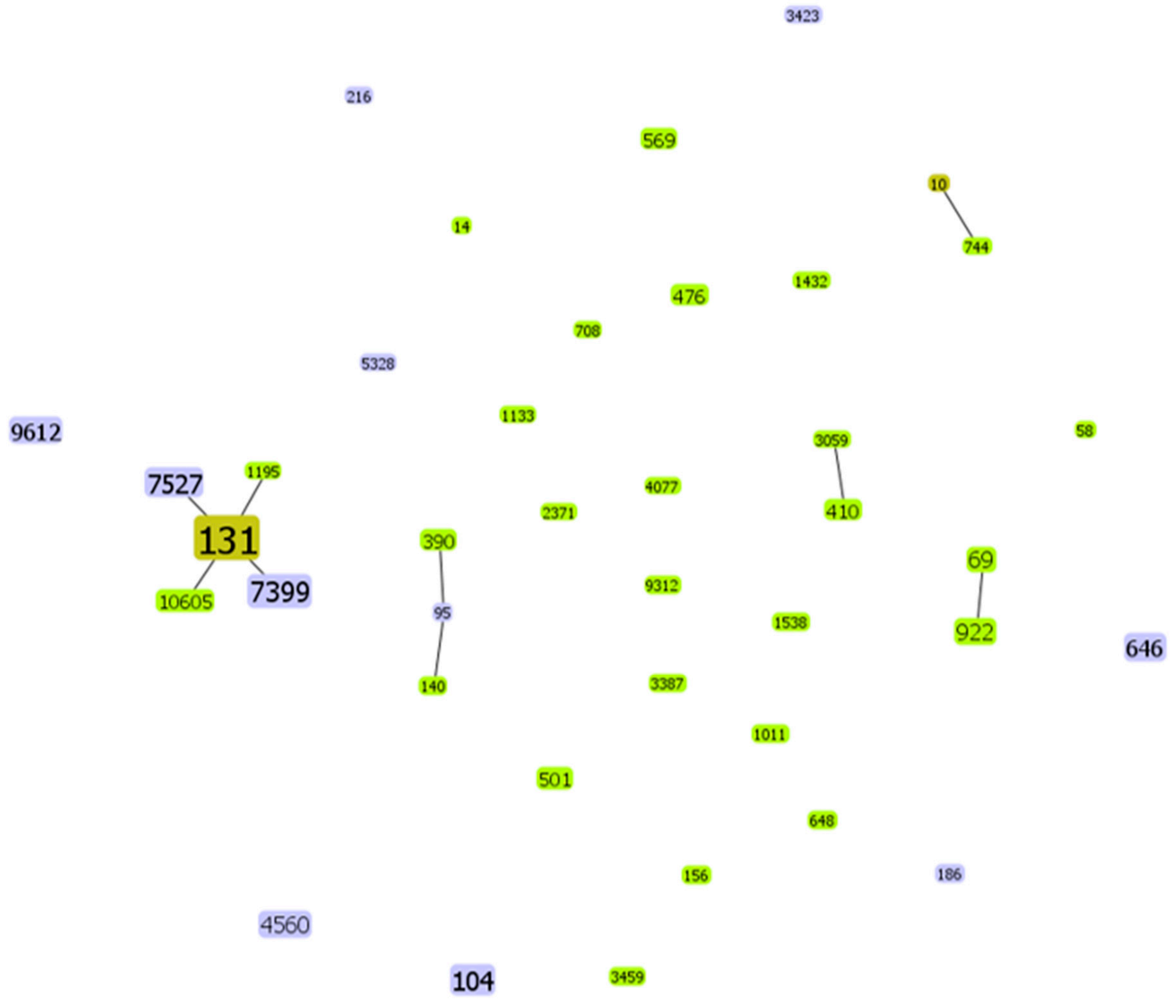

Figure S1. An eBURST diagram showing the relationships between the detected sequence types of *E. coli* isolates included in this study.
